# TT3D: Leveraging precomputed protein 3D sequence models to predict protein–protein interactions

**DOI:** 10.1093/bioinformatics/btad663

**Published:** 2023-10-28

**Authors:** Samuel Sledzieski, Kapil Devkota, Rohit Singh, Lenore Cowen, Bonnie Berger

**Affiliations:** Computer Science and Artificial Intelligence Laboratory, Massachusetts Institute of Technology, Cambridge, MA 02139, United States; Department of Computer Science, Tufts University, 177 College Avenue, Medford, MA 02155, United States; Department of Biostatistics & Bioinformatics, Duke University, Durham, NC 27705, United States; Department of Cell Biology, Duke University, Durham, NC 27705, United States; Department of Computer Science, Tufts University, 177 College Avenue, Medford, MA 02155, United States; Computer Science and Artificial Intelligence Laboratory, Massachusetts Institute of Technology, Cambridge, MA 02139, United States; Department of Mathematics, Massachusetts Institute of Technology, 77 Massachusetts Avenue, Cambridge, MA 02139, United States

## Abstract

**Motivation:**

High-quality computational structural models are now precomputed and available for nearly every protein in UniProt. However, the best way to leverage these models to predict which pairs of proteins interact in a high-throughput manner is not immediately clear. The recent Foldseek method of van Kempen *et al.* encodes the structural information of distances and angles along the protein backbone into a linear string of the same length as the protein string, using tokens from a 21-letter discretized structural alphabet (3Di).

**Results:**

We show that using both the amino acid sequence and the 3Di sequence generated by Foldseek as inputs to our recent deep-learning method, Topsy-Turvy, substantially improves the performance of predicting protein–protein interactions cross-species. Thus TT3D (Topsy-Turvy 3D) presents a way to reuse all the computational effort going into producing high-quality structural models from sequence, while being sufficiently lightweight so that high-quality binary protein–protein interaction predictions across all protein pairs can be made genome-wide.

**Availability and Implementation:**

TT3D is available at https://github.com/samsledje/D-SCRIPT. An archived version of the code at time of submission can be found at https://zenodo.org/records/10037674.

## 1 Introduction

Experimental protein–protein interaction (PPI) data remain sparse in most model organisms and even more so in other species. Recent deep learning methods that predict PPIs solely from sequence seek to address this limitation. In prior work, we introduced D-SCRIPT (Sledzieski *et al.* 2021) and Topsy-Turvy (Singh *et al.* 2022), two deep learning methods that rapidly predict whether two proteins will physically bind in the cell using only protein sequence information. We call these methods lightweight deep-learning methods, since they are computationally efficient enough to be run genome-wide. These methods can be contrasted with classical PPI docking methods (Porter *et al.* 2019) that require different inputs (namely the 3D structures of the proteins), and also produce different outputs (in addition to predicting *if* the proteins bind, they also model *how* they bind).

The advent of large deep learning methods for structure prediction like OmegaFold (Wu *et al.* 2022), AlphaFold2 (Jumper *et al.* 2021), ESMFold (Lin *et al.* 2023), and RoseTTAFold (Baek *et al.* 2021), however, mean that high-quality 3D protein structural models can now be produced when only protein sequence is available as input. While these methods are too expensive to run from scratch at genome-wide scale, thanks to large community-wide efforts, there is no longer a need to run them from scratch: high quality computational structural models are now being made publicly available for nearly every protein in UniProt (Varadi *et al.* 2022, Burley *et al.* 2023). In this work, we ask how this wealth of computational work and high-quality predicted structural information can be re-used to improve lightweight deep-learning methods that rapidly predict whether two proteins will physically bind in the cell. One potential approach is to run computational fold-and-dock methods such as AlphaFold-Multimer (Evans *et al.* 2021, Zhu *et al.* 2023), or full complex structure prediction (Weissenow *et al.* 2022, Burke *et al.* 2023, Harini *et al.* 2023, Lensink *et al.* 2023). While these approaches are powerful for a small set of candidate pairs, they are still too computationally expensive to scale genome-wide, e.g. to create a full predicted PPI atlas for a nonmodel organism.

However, the wide availability of protein structure prediction methods has also coincided with breakthroughs in compact representation of protein structure and structure search. One such example is Foldseek (van Kempen *et al.* 2023), which uses a vector-quantized variational autoencoder (VQ-VAE) (Van Den Oord *et al.* 2017) to encode a protein structure as a sequence of discrete embedding vectors, each of which is then mapped onto a set of characters which called the 3D interaction alphabet (3Di). This process maps the 3D space of protein structure into a single dimensional 3Di sequence, which can then be used with fast sequence search tools such as BLAST (Altschul *et al.* 1997) or MMseqs2 (Steinegger and Söding 2017) to identify structurally similar proteins (Barrio-Hernandez *et al.* 2023).

Here, we introduce Topsy-Turvy 3D (TT3D), which builds off of prior work in sequence-based PPI prediction (Singh *et al.* 2022) to incorporate structure by jointly modeling both amino acid sequence and 3Di sequence. We demonstrate that TT3D is able to take advantage of the compact representation of protein structure to improve the accuracy of PPI prediction in a cross-species context. In an era where high-quality predictions of protein structure are readily available for many proteins, we expect that TT3D can be easily substituted into pipelines which use lightweight sequence-only deep learning prediction methods to make high-quality predictions, while remaining fast enough to be applied at genome scale.

## 2 Materials and methods

**Figure 1.**
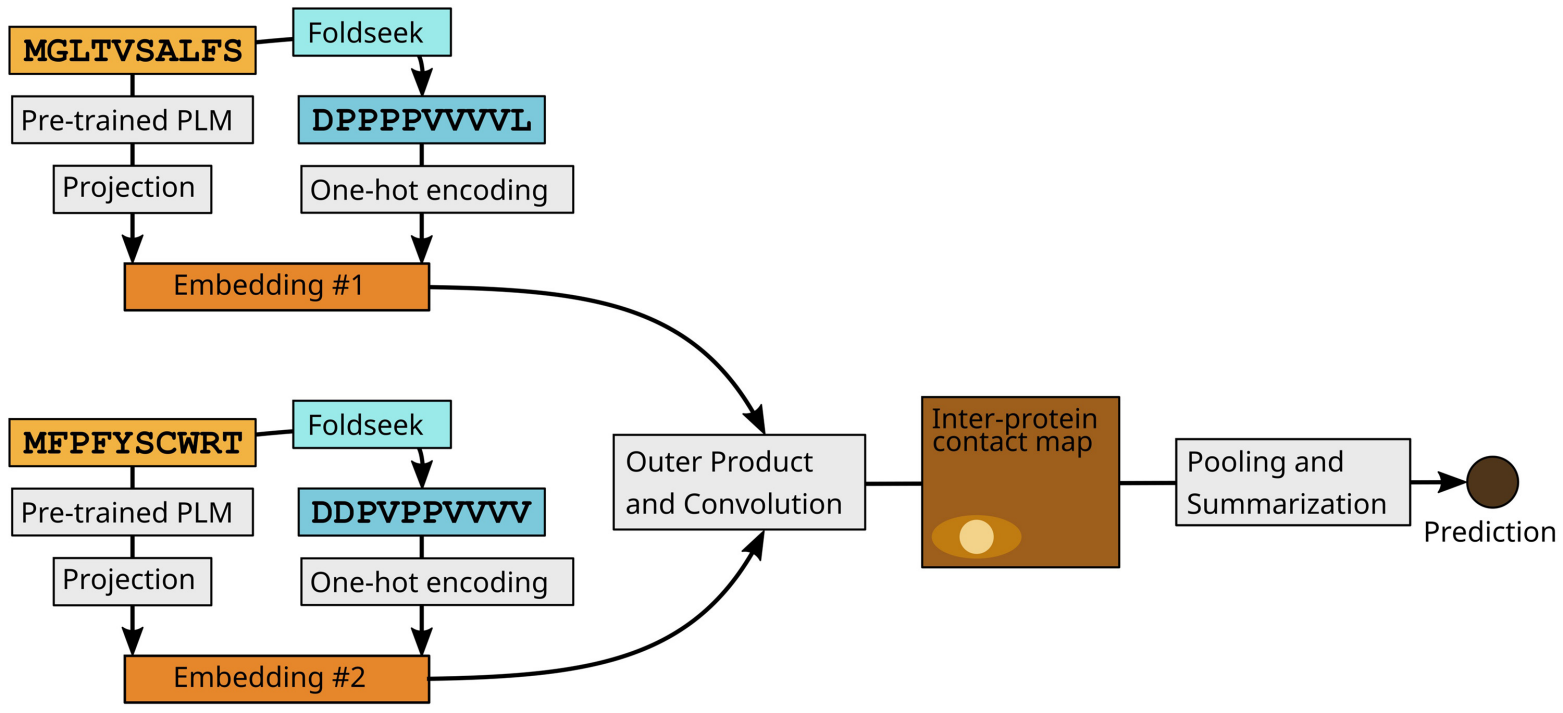
TT3D model architecture. TT3D follows the structure and training procedures of Topsy-Turvy, but with an augmented protein embedding. We concatenate a one-hot encoding of the Foldseek 3Di (van Kempen *et al*. 2023) string to the protein language model (PLM)-based embedding before passing this representation into the convolutional portion of the architecture.

TT3D augments the inputs to the basic Topsy-Turvy architecture with encodings of the Foldseek-generated 3Di sequence (see Fig. 1) (van Kempen *et al.* 2023). In Topsy-Turvy, the amino acid sequence $x=x_{1}x_{2}\ldots x_{n}$ is numerically encoded using the Bepler & Berger protein language model (Bepler and Berger 2019, 2021) as $X\in R^{n\times 6165}$, which is then reduced in dimension via a multi-layer perceptron to a projection $X^{*}\in {\mathbb{R}}^{n\times 100}$.

In TT3D, we additionally convert the protein sequence *x* to a 3Di sequence $y=y_{1}y_{2}\ldots y_{2}$ using Foldseek. If a crystal structure is available for the protein, Foldseek can be directly applied. If the sequence is not available in the PDB, we query for an exact match for it in AlphaFoldDB (Varadi *et al.* 2022), and this structure is then used for extraction of the 3Di sequence by Foldseek. If no such hit can be found, we conservatively add an uninformative all-X 3Di sequence. We represent *y* with a one-hot encoding, yielding $Y\in R^{n\times 21}$. We then concatenate the embeddings from the language model and from Foldseek, resulting in a joint embedding $E=[X^{*};Y]\in {\mathbb{R}}^{n\times 121}$. Given two protein sequences $x_{1},x_{2}$, we combine embeddings $E_{1},E_{2}$ as in D-SCRIPT and Topsy-Turvy (Sledzieski *et al.* 2021, Singh *et al.* 2022) to predict a probability of interaction. The Topsy-Turvy loss function is used to train the model using back-propagation.

## 3 TT3D outperforms state-of-the-art deep learning-based methods

We evaluate TT3D in the same cross-species setting where D-SCRIPT and Topsy-Turvy were originally tested. Following (Sledzieski *et al.* 2021), TT3D was trained and validated on known human PPI from the STRING database (Szklarczyk *et al.* 2021), filtered for experimentally determined physical binding interactions.

Then, the best model trained on human PPIs was tested on known interactions from other model organisms such as mouse (*Mus musculus*), fly (*Drosophila melanogaster*), roundworm (*Caenorhabditis elegans*), *Escherichia coli*, and brewer’s yeast (*Saccharomyces cerevisiae*), also from STRING. Sequences were clustered with human sequences at 40% similarity using CD-HIT (Li and Godzik 2006) and those with high similarity to proteins in the training set were removed. We measure model performance using the area under the precision–recall curve (AUPR). The test sets were constructed to have a 1:10 ratio of positives to negatives, so a random method would have an AUPR of 1/11≊0.09.

**Figure 2.**
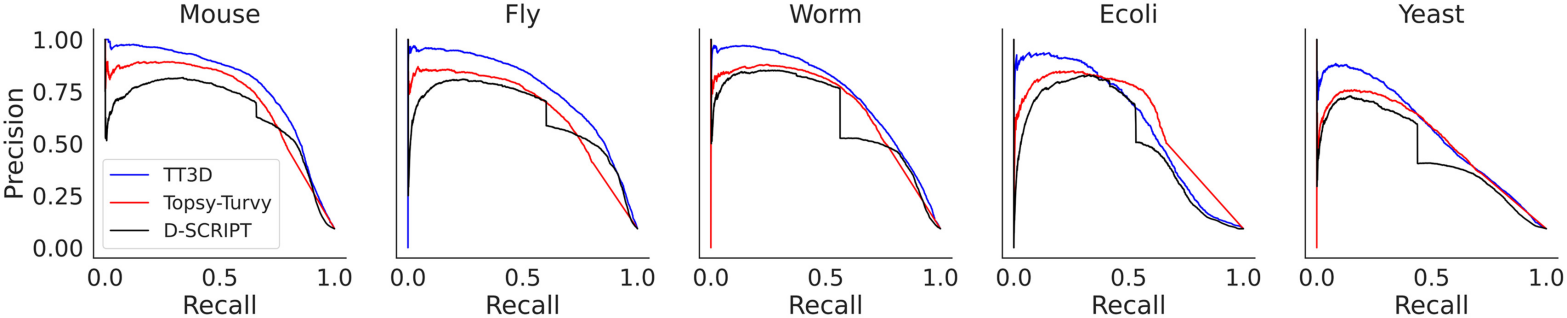
Precision–recall curves for TT3D, Topsy-Turvy, and D-SCRIPT. Our experiments in organisms: Mouse, Fly, Roundworm, *E.coli*, and Brewer’s Yeast show TT3D significantly outperforming the other methods while predicting unknown PPI interactions.

We compare TT3D to D-SCRIPT and Topsy-Turvy, neither of which incorporate structural information, and find that augmenting the Topsy-Turvy model with the encoded 3Di Foldseek sequence improves its PPI predictions. We also test against simple sequence and structure homology-based approaches. In Fig. 2, we show precision–recall curves for each of the three deep learning methods on the five benchmark test sets. TT3D performs significantly better than the other methods for all organisms that we tested on. In addition to overall performance, early precision (i.e. precision at low recall) is important, because often only a small number of highly predicted interactions are selected for downstream experimental prediction. We find that the precision values at low recall are closer to 1 for TT3D, which indicates that its top predictions are much more accurate than both D-SCRIPT and Topsy-Turvy.

## 4 Comparing TT3D’s performance with simple sequence and structure homology transfer approaches

Sequence or structure-based homology approaches can also be used to transfer PPI annotations across species. We benchmarked TT3D against two such approaches, one based on Ensembl-provided sequence homology (Smedley *et al.* 2009), and the other based on structural homology inferred using a pipeline of AlphaFoldDB, Foldseek, and MMseqs2 (see Supplementary Materials Online). We note two major challenges with such annotation transfer approaches. First, due to the bias in how candidate PPIs were chosen for assays, just knowing that a pair of target proteins have human homologs turns out to be a surprisingly good predictor of their interaction, achieving precision (recall) of 0.2065 (0.658) and 0.2313 (0.4442) in fly and yeast, respectively. Second, such an approach does not provide a probability of an interaction, so neither an average precision nor precision–recall curve can be computed. Nonetheless, we compared TT3D to these approaches by generating the exhaustive set of fly (or yeast) PPI candidates by considering all possible transfers of human PPIs and scored these against ground-truth PPIs. TT3D outperformed both sequence and structure-based annotation transfer, achieving about 5× and 17× greater precision in fly than sequence and structure-based approaches, respectively (see Supplementary Materials for detailed precision–recall metrics).

## 5 Availability and implementation

For inference with TT3D, as well as with Topsy-Turvy and D-SCRIPT, we make available a web interface at https://cb.csail.mit.edu/cb/dscript/. This interface is implemented with Gradio (Abid *et al.* 2019) and hosted on HuggingFace spaces, and allows the user to upload a .fasta formatted file with sequences and a .tsv file with candidate protein pairs, and get back predictions for the desired model. This interface additionally leverages 3Di sequences from (Heinzinger *et al.* 2023).

For model training or larger-scale inference from the command line, TT3D is implemented in Python 3 as part of the dscript package for predicting PPIs, which is available from the PIP package repository (pip install dscript) or on GitHub at https://github.com/samsledje/D-SCRIPT. Model training and inference was performed on a machine with a 112-core Intel Xeon Gold 6258R CPU and using a single NVIDIA A100 GPU. TT3D is trained for a maximum of 10 epochs, and the best performing model in cross-validation is used for making predictions. We make the trained model available to download at https://d-script.readthedocs.io/en/stable/, where it can be used to make new predictions with the dscript predict command.

TT3D requires that Foldseek (van Kempen *et al.* 2023) be installed and that 3Di sequences be generated for protein sequences in the training or inference set. Structures in .pdb format must be available for all sequences, either natively or generated by a structure-prediction method such as OmegaFold (Wu *et al.* 2022), AlphaFold2 (Jumper *et al.* 2021), or RoseTTAFold (Baek *et al.* 2021). Foldseek can be downloaded and build from source on Github at https://github.com/steineggerlab/foldseek. For convenience, we provide the command dscript extract-3Di, which uses the user’s installed Foldseek to translate a set of structures into a .fasta file containing 3Di sequences.

To run TT3D, users should run the command dscript train – allow_foldseek, where – allow_foldseek is an optional command that runs the training iterations in “Foldseek” mode. While running in this mode, the user should provide the corresponding 3Di sequences in .fasta format using the – foldseek_fasta argument.

## Supplementary Material

btad663_Supplementary_Data
