# Supplementary material for: TT3D: Leveraging precomputed protein 3D sequence models to predict protein–protein interactions
Source: Bioinformatics. 2023 Oct 28;39(11):btad663. doi: 10.1093/bioinformatics/btad663 (PMC10640393; doi:10.1093/bioinformatics/btad663)
Supplement: btad663_Supplementary_Data [file btad663_supplementary_data.pdf]

Supplementary Materials

Comparison of TT3D to annotation-transfer techniques

To investigate the performance of homology-only mapping on protein-protein interaction, we identified sequence- and structure-based homologs in fly and yeast. To determine sequence homologs, We used Ensembl-provided homology mappings. We originally considered, but then rejected the use of only one-to-one mappings, i.e., where a single fly protein is mapped to just one human protein, which yielded only 68 mappings between human and fly PPIs. Our subsequent analyses therefore were in the many-to-many homology mapping regime. To determine structure homologs, we used structures from AlphaFold DB, obtained the 3Di sequence representations from Foldseek, and then applied MMSeqs2 (with an e-value threshold of 10^{-10}) to map structural homologs between fly (or yeast) and human.

We took the entire set of human PPIs from our training set and mapped them to all potential fly (or yeast) homolog pairs, reasoning that this would be the exhaustive set of potential fly PPIs that any annotation transfer method could potentially consider as true. We note that this mapping resulted in a massive expansion of potential fly PPIs. The $\sim$ 38,000 human training-set PPIs were mapped to $\sim$180,000 fly PPIs (Ensembl-based homology mapping) and $\sim$3.1 million fly PPIs (structure-based homology mapping). We considered the union of these with fly pairs with the true positive fly pairs ($\sim$27,000 from STRING v11; physical binding interactions only). We created analogous networks for yeast, observing that the set of homologs between human and yeast is smaller. Because of distinct sequence and structure-based homology mapping schemes, these evaluations were essentially on two separate datasets, which we denote as "Networks from Sequence Homology" and "Networks from Structure Homology", respectively.

We applied TT3D on these datasets, computing its precision and recall curves. For homology-based annotation transfer, precision was computed as standard: Intersection(*True_Positives, Predicted_Positives*)/ *Predicted_Positives*. Here, *True_Positives* are the ground-truth fly PPIs while *Predicted_Positives* is the set of PPIs mapped from human. Recall was similarly computed as per its standard definition. We note that annotation transfer provides only one set of precision and recall scores, while TT3D's score (in the range 0–1) can be thresholded to provide a range of precision-at-desired-recall scores and the corresponding Areas Under Precision-Recall Curve.

**Experiment**	**Species**	**Predictions By**	**Recall**	**Precision**	**AUPR**
-	Fly	TT3D	-	-	0.6263
-	Yeast	TT3D	-	-	0.4755
Homology Only	Fly	Sequence	0.658	0.2065	-
Homology Only	Fly	Structure	0.7864	0.2967	-
Homology Only	Yeast	Sequence	0.4442	0.2312	-
Homology Only	Yeast	Structure	0.8438	0.1386	-
Homology+Interaction in Human	Fly	Sequence	0.384	0.99	-
Homology+Interaction in Human	Fly	Structure	0.6396	0.7894	-
Homology+Interaction in Human	Yeast	Sequence	0.1922	0.9688	-
Homology+Interaction in Human	Yeast	Structure	0.5126	0.4879	-
Networks from Sequence Homology	Fly	TT3D	0.3858	0.2934	0.2812
Networks from Sequence Homology	Fly	Sequence	0.3858	0.0598	-
Networks from Sequence Homology	Yeast	TT3D	0.1917	0.6913	0.7409
Networks from Sequence Homology	Yeast	Sequence	0.1917	0.4025	-
Networks from Structure Homology	Fly	TT3D	0.6452	0.0604	0.0759
Networks from Structure Homology	Fly	Structure	0.6452	0.0056	-
Networks from Structure Homology	Yeast	TT3D	0.5397	0.0856	0.0583
Networks from Structure Homology	Yeast	Structure	0.5397	0.0131	-
